# Early disruptions in vitamin D receptor signaling induces persistent developmental behavior deficits in zebrafish larvae

**DOI:** 10.1371/journal.pone.0335156

**Published:** 2025-11-14

**Authors:** Morgan Barnes, Derek Burton, Kurt Marsden, Seth W. Kullman

**Affiliations:** 1 Department of Biological Sciences, North Carolina State University, Raleigh, North Carolina, United States of America; 2 Toxicology Program, North Carolina State University, Raleigh, North Carolina, United States of America; 3 Center for Human Health and the Environment. North Carolina State University, Raleigh, North Carolina, United States of America; Radboud University Medical Centre, NETHERLANDS, KINGDOM OF THE

## Abstract

A critical function of the nervous system is to rapidly process sensory information and initiate appropriate behavioral responses. Defects in sensory processing and behavior selection are commonly observed in neuro-psychiatric conditions including anxiety, autism (ASD), and schizophrenia. The etiology of sensory processing disorders remains equivocal; however, it is hypothesized that extrinsic environmental factors can play fundamental roles. In this study we examine the importance of vitamin D (1α, 25-dihydroxyvitamin D3) receptor signaling during early life stage development on sensory processing and neurobehavioral health outcomes. While vitamin D has traditionally been associated with mineral ion homeostasis, accumulating evidence suggests non-calcemic roles for vitamin D including early neurodevelopment. Here we demonstrate that systemic disruption of vitamin D receptor (VDR) signaling with a conditional dominant negative (dnVDR) transgenic zebrafish line results in specific visual and acoustic sensorimotor behavior defects. Induction of dnVDR between 24–72 hours post fertilization (hpf) resulted in modulation of visual motor response with demonstrated attenuation in acute activity and hypolocomotion across multiple swimming metrics when assayed at 6- and 28-days post fertilization (dpf). Disruption in VDR signaling additionally resulted in a strong and specific attenuation of the Long-Latency C-bends (LLC) within the acoustic startle response at 6 dpf while Short-Latency C-bends (SLC) were moderately impacted. Pre-pulse inhibition (PPI) was not impacted in young larvae, however young adult fish exhibited a significantly attenuated PPI at 28 dpf suggesting an inability to properly modulate their startle responses and persistent effects of VDR modulation during early development. Overall, our data demonstrate that modulation of vitamin D signaling during critical windows of development irreversibly disrupts the development of neuronal circuitry associated with sensory processing behaviors which may have significant implications to neurobehavioral health outcomes.

## Introduction

Neuropsychiatric disorders such as anxiety, autism (ASD), and schizophrenia (SZ) typically have defects in sensory processing and behavior [1]. The nervous system is critical for processing sensory information and initiating appropriate behaviors. Despite the biological and clinical relevance, our understanding of the cellular and molecular mechanisms regulating these processes is limited, in part due to the intricate and dynamic circuitry involved in complex human decision-making. The origins of sensory processing disorders are complex, as intrinsic/genetic, extrinsic/environmental and the interactions of intrinsic and extrinsic factors can play fundamental roles [2]. Low levels of vitamin D during early stages of development have been linked to various brain disorders such as schizophrenia and autism with associated deficits in sensory processing [3]. However, to date, there remain critical gaps in the knowledge of how vitamin D deficiency (VDD) contributes to neurobehavioral health outcomes.

Vitamin D was first identified with the investigation of rickets by McCollum who coined the term vitamin D [4]. Subsequently it was demonstrated that vitamin D is essential for dietary calcium absorption and skeletal mineralization [5]. Research has since illustrated that vitamin D serves many physiological roles and vitamin D deficiency is now associated with select disease etiologies including cardiovascular disease, cancer, stroke, metabolic disorders and neurodegenerative disease [6,7].

Vitamin D is a fat-soluble vitamin that can be obtained through the diet or UVB conversion of 7-dehydrocholesterol to pre-vitamin D3 in the skin followed by metabolic activation to vitamin D3. There are multiple forms that circulate throughout the body including the inactive 25 hydroxyvitamin D, (25(OH)D) and the active form 1,25-hydroxyvitamin D (1,25(OH)2D). 1,25-hydroxyvitamin D is formed through sequential hydroxylations involving both 25-hydroxylase and 1α-hydroxylase [8]. The active form of vitamin D serves as a potent ligand for the vitamin D receptor (VDR) and together with its obligate heterodimerization partner RXR, this receptor pair facilitates both transactivation and trans-repression of genes critical to select cellular processes including maintenance of calcium and phosphate homeostasis, cell proliferation, immune system function, cardiovascular function, and neurodevelopment [8].

Vitamin D deficiency is defined as 1,25-hydroxyvitamin D levels lower than 50 nmol/L or 20 ng/mL and multiple factors can affect vitamin D levels including age, lifestyle, skin pigmentation, exposure to environmental agents, diseases that affect the intestinal tract and people who have undergone gastric bypass surgery [8,9]. About a billion people worldwide are vitamin D deficient (VDD) or insufficient, making this a global issue [10]. The prevalence of VDD in pregnant women, who are especially vulnerable, can be as high as 50% globally if not more [11]. Pregnant women are at particularly high risk for VDD, due to increased demand for vitamin D to supplement the growing fetus, which is associated with indirect and direct adverse neurodevelopmental effects [11]. The vitamin D receptor (VDR) itself has been found to be expressed within multiple regions of the mammalian brain in conjunction with 1α-hydroxylase, an essential enzyme necessary to convert inactive vitamin D to the active form [12]. Multiple studies indicate a pivotal role for vitamin D as a neurosteroid in the brain mediating essential neural functions including regulation of calcium homeostasis [13], deposition of beta-amyloid [14,15], regulation of oxidative stress [16,17], inflammation [17,18], cell proliferation [19,20], cell differentiation [19,21], regulation of neurotropic factors [22–25] and biosynthesis of neurotransmitters [26–29]. These actions have categorized vitamin D as a neuroprotectant and has identified vitamin D as being critical to brain health [3,6].

Translational models including zebrafish (*Danio rerio*) have proved a valuable resource to investigate developmental neuroscience research. The zebrafish genome is well annotated, and it has been demonstrated that zebrafish share about 70% genetic homology to humans [30]. Additionally, zebrafish possess similar neurochemistry to humans including neurotransmitter receptors, transporters and synthesizing enzymes [30]. The zebrafish central nervous system (CNS) begins to be specified as early as 6 hours post fertilization (hpf), at the beginning of gastrulation [30]. At approximately one week old, zebrafish brains are estimated to contain roughly 100,000 neurons and can perform many complex behaviors despite being so young [31]. Zebrafish have been demonstrated to be similar to mammalian research models via brain macro-organization and cellular morphology [32]. CNS structures in zebrafish can be identified as early as 10 hpf, and by 24 hpf the primary divisions of the brain, the forebrain, midbrain and hindbrain, are defined by morphogenetic boundaries. Additionally, zebrafish larvae possess most of the major neurotransmitter’s receptors, transporters and enzymes [32]. Zebrafish as young as 2 days post fertilization (dpf) are able to respond to external stimuli through sensorimotor integration in the CNS [30]. Zebrafish have a very social nature and are diurnal animals that rely on vision, similar to most humans [32]. Over the past several decades multiple assays have been developed to index a broad range of zebrafish behaviors including scoot swimming; burst swimming; routine turns; stimulus-specific turning responses such as J-, C-, and O-bend turns; optokinetic and optomotor responses; prey tracking; phototaxis; thigmotaxis; escape and avoidance behaviors; non-associative learning; and visual recognition memory [33,34]. These assays have proven to be sensitive to the early and persisting impacts of exogenous drug/toxicant exposures, dietary modifications and/or genetic manipulations [35,36]. These tests provide a powerful approach to assess alterations within early neurodevelopment and sensory processing, with defects reflecting dysfunction of the underlying cellularity and/or neural circuits.

In this study we take advantage of a set of well-established behavioral assays to investigate how modulation of developmental VDR signaling modulates larval zebrafish sensory processing. Specifically, we use an inducible dominant-negative VDR transgenic line to simulate VDD by limiting embryonic/larval VDR signaling, and we show that VDR signaling is critical within a defined developmental window for normal responses to both visual and auditory stimuli. Furthermore, we find that some of these behavioral changes persist into young adulthood, and they are consistent with changes in the expression of genes in several key neural signaling pathways.

## Methods

### Fish husbandry and maintenance

Zebrafish (Danio rerio) were housed and cared for according to standard protocols approved by the North Carolina State University (NC State) Institutional Animal Care and Use Committee (#22–378). Adult zebrafish were maintained at appropriate densities in 9L tanks as part of a recirculating aquatics system under a 14:10hr light: dark cycle. Water temperature was maintained at 28.5 ± 0.5°C with a pH between 6.8 and 7.5. Two strains of zebrafish (Danio rerio) were used in this study including the wildtype strain EK and a heat shock inducible vitamin D receptor dominant negative transgenic strain tg(hsp:dnVDRa:BFP) obtained from the Poss lab, Duke University [37]. For embryo collection, mature zebrafish (4–12 months) were bred under standard conditions and viable embryos were sorted and collected. Embryos were maintained in petri dishes with E3 media in an incubator set to 28 ± 0.5 °C. Embryo media was changed daily. All fish were monitored daily to ensure there was no change in health to prevent any suffering. Heat shock exposure did not result in any physical harm to the animals and no fish had to be sacrificed due to a decline in health and or increased stress from the heat shock exposures. Additionally, none of the assessed endpoints induced any harm to any fish. Upon completion of each experiment, fish were euthanized using ice water at indicated time points for RNA collection. To reduce the number of fish utilized, we used the same cohort of fish to assess behaviors at 6 dpf and 28 dpf.

### Heat shock induction

Heat shock was performed on zebrafish embryos at either 24, 48 or 72 hours post fertilization (hpf) using a thermocycler. Heat shock was induced at indicated times by placing embryos/larvae in a 96 well plate, 1 fish per well, with E3 media then incubated in a thermocycler at 38°C for 30 minutes. Embryos/larvae were subsequently screened 5–8 hours post heat shock for induction of blue fluorescent protein (BFP) expression using a UV filter on an epi-fluorescent (Nikon Eclipse TE2000-S) microscope, and fish were sorted into two cohorts according to the presence (+) or absence (-) of BFP expression which is linked to dnVDR through the P2A sequence.

### Acoustic startle response

Vibro-acoustic startle responses were assayed using custom built behavioral systems at NC State. We exposed 6 dpf larvae to 10 trials at each of 6 intensities of acoustic stimuli (ranging from 10–60 dB) at a 20 s inter-stimulus interval (ISI) for a total of 60 stimuli. To assess pre-pulse inhibition (PPI), larvae were exposed to 10 pairs of a weak (23 dB) pre-pulse stimuli and strong (60 dB) stimuli. There was a 300 ms break between the pre-pulse and pulse, and a 20 s ISI between pairs of stimuli. For 28 dpf fish, which habituate more readily than younger fish, we ran 5 trials at each of 3 intensities, with a 2 min ISI to assess startle responsiveness, followed by 10 PPI trials. We captured responses with a Photron Mini-UX50 high-speed camera at 1000 frames/s. To evaluate movement kinematics (response frequency, latency, turning angle) we used the FLOTE software package [38]. For each phase of the assay, we measured multiple endpoints, including response frequency, latency, turning angles, angular velocity, duration and distance traveled. C1 (initial C-bend) and C2 (counter bend) angles are measured as the maximum change in head angle in reference to the initial starting position (for C1) or the end of C1 (for C2). Duration is calculated as the time elapsed during the first movement sinusoid (C1) or second movement sinusoid (C2). A minimum of 20 larvae were tested for each replicate set, with three replicate analyses performed.

### Zebrafish larval visuo-motor response (VMR) assay

The larval VMR behavioral analysis was conducted using the Noldus video-surveillance system and animal movement tracking software EthoVision XT (Noldus Information Technology, The Netherlands). All larval testing was run between 9:00 AM and 1:00 PM to ensure consistency with circadian cycles. Larval locomotion assays were conducted using a DanioVision™ lightbox running EthoVision XT® tracking software (Noldus Inc., Wageningen, The Netherlands). Heat shocked, 6- day or 28-day old larvae were transferred to multi-well plates, with all experimental groups represented on each plate and across multiple plates. Following a 1-hour incubation at 28°C, locomotor activity is tracked during an initial 30-min acclimation period in the dark (0% illumination), followed by five cycles of 10 min at 100% illumination (5000 lx) and 10 min at 0% illumination. An infrared camera captures larval locomotion across the programmed cycles. For each trial the average distance moved, total velocity, activity state, and distance to point is determined for each subject. The raw data of movement per minute for the alternating 10-min dark and light phases of the session was utilized for the statistical analyses and a minimum of 20 larvae was tested for each replicate set, with three replicate analyses performed.

### Epifluorescent imaging of BFP expression

To localize BFP expression, post heat shock tg(hsp:dnVDRa:p2a:BFP) zebrafish were anesthetized using 0.006% tricaine then placed in a transverse position for imaging. Images were collected using Elements Software and camera.

### RNA isolation

Larval zebrafish (6 dpf) were euthanized in ice water. 15–20 whole larvae we subsequently pooled per RNA sample with 4 replicated samples. Samples were immediately flash frozen in liquid nitrogen and tissues were homogenized in TRI Reagent® (Ambion®, Life Technologies, Carlsbad, CA, USA) using a handheld BioVortexer (Thomas Scientific, Swedesboro, NJ, USA). Total RNA was isolated according to the TRI Reagent manufacturer’s protocol. Total RNA was quantified using the Qubit® RNA HS Assay Kit (Invitrogen™, USA) containing Qubit® RNA HS Reagent and Buffer with the Qubit® 3.0.

### Quantitative real-time PCR (qPCR)

Quantitative Real-Time PCR (qPCR) was used to measure targeted gene expression in whole 6 dpf larval zebrafish. cDNA was synthesized from 1 µg total RNA using 10x random primers, 10x reverse transcription buffer, MultiScribe Reverse Transcriptase, and 10mM deoxynucleotide triphosphates from a High-Capacity cDNA Reverse Transcription Kit (Applied Biosystems, Foster City, CA) along with RNasin(R) RNase Inhibitor (Promega, Madison, WI). Primer sequences were designed using Primer3web, version 4.1.0. Primers were ordered from Integrated DNA Technologies, Inc. (Coralville, Iowa). See (S1 Table) for a full list of primer sequences. Gene expression patterns were quantified using a QuantStudio 3 real time PCR machine. Biological replicates (n = 3–4/group) were plated in triplicates and amplified in a 96-well, clear Olympus PCR plate (Genesee, Morrisville, NC). Each well contained a 20 µL mixture of Ultrapure water (Invitrogen, Marietta, OH), forward primer, reverse primer, cDNA, and iTaq Universal SYBR Green Supermix (Bio-Rad, Hercules, CA). The plate was then covered with an adhesive seal and spun down in a centrifuge. Each reaction occurred under the following conditions: (1) 50 °C for 2 min, (2) 95 °C for 10 min, (3) 95 °C for 15 sec followed by 60°C for 1 min (repeated 40 times). This cycle was followed by a dissociation stage which ensured primer specificity and confirmed the absence of primer dimerization: (4) 95 °C for 15 secs 60 °C for 1 min, 95 °C for 15 sec, 60 °C for 1 min. Individual threshold cycle values (Ct) were determined for each reaction by the QuantStudio 3 Software and relative fold change differences for each gene across each sample was calculated according to the ΔΔCt method [39]. Gene expression was normalized to *efla* as the reference gene.

### Statistical analysis

All statistical analysis was conducted using Graphpad 10.5. Data sets were tested for “normality” utilizing the D’Agostino & Pearson, Anderson-Darling, Shapiro-Wilk and Kolmogorov tests representing Normal (Gaussian) distribution. Data that failed 2 or more of those tests was considered not normal. For normal data- statistical inferences was conducted using either an unpaired t-test or for data with significant differences in variances as determined by F test, a Welch’s t-test was utilized. Data that did not have a “normal” distribution was assessed using an unpaired experimental design with a nonparametric Mann-Whitney t-test. Statistical analyses were based on pair wise comparisons within the same heat shock time period (i.e., 24- vs 24+).

## Results

### Heat shock induction of dnVDR:BFP

Induction of heat shock at 24, 48, 72 hpf induced robust BFP expression in tg(hsp:dnVDR:BFP) zebrafish larvae as observed under epifluorescence. A representative image of BFP expression is shown in (S1 Fig). BFP expression depicted in our cohort was comparable to BFP expression shown in previous studies with strong localization in the brain [37]. The weak peptide bonds within the p2A linker allows for BFP to mark the cells in which dnVDR is expressed without interfering with its function. Post heat shock zebrafish larvae were observed under bright field microscopy to ensure heat shock treatment did not result in any observable morphological alterations to zebrafish larvae.

### Induction of dnVDR modulates early life stage visual motor response (VMR) behavior

**Fig 1:**
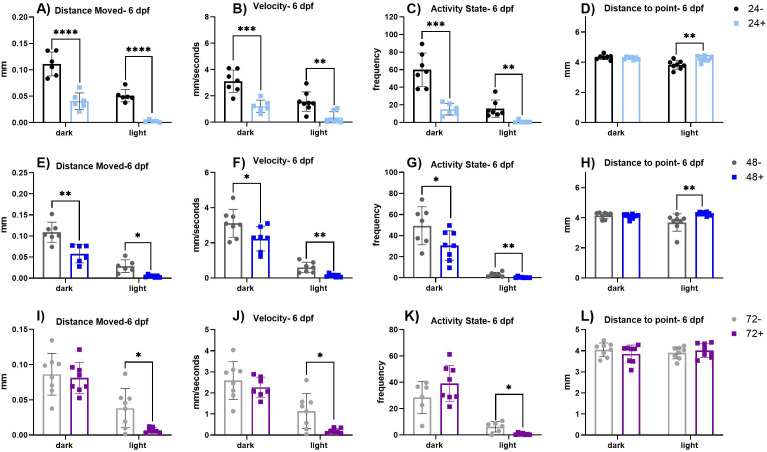
Parameters of VMR separated by light or dark periods run at 6 dpf. (A-D) VMR parameters in dnVDRa induced zebrafish at 24 hpf. There is a significant decrease in distance moved in the dark (p < 0.0001) and light periods (p < 0.0001), a decrease in velocity in the light (p < 0.001) and dark periods (p < 0.01), a decrease in activity state in the light (p < 0.01) and dark periods (p < 0.001) and an increase in distance to point in the light period (p < 0.01) in the 24 + fish. (E-H) VMR parameters in dnVDRa induced zebrafish at 48 hpf. There is a significant decrease in distance moved in the dark (p < 0.01) and light (p < 0.05) periods, a significant decrease in velocity in the dark (p < 0.05) and light (p < 0.01) periods, a significant decrease in activity state in the dark (p < 0.05) and light (p < 0.01) periods and a significant increase in distance to point in the light period (p < 0.01) in the 48 + fish. (I-K) VMR parameters in dnVDRa induced zebrafish at 72 hpf. There is a significant decrease in distance moved (p < 0.05), a significant decrease in velocity the light period (p < 0.05) and a significant decrease in activity state in the light periods (p < 0.05) in the 72 + fish. Values represent the mean by separate measurements of 6-8 fish/group. Error bars represent the standard deviation of each group. All groups passed Normality tests except for the following: 24- & 24+ (activity state-light), 48+ (activity state-light), 48- (distance to point-dark & light) and 72+ (distance to point-dark). For any group that failed Normality tests, a Mann-Whitney t-test was utilized. An unpaired t-test was used for the rest of the groups except for when variances were significantly different (distance moved- light: all groups, velocity-light: 48 & 72, activity state- dark: 24, activity state-light: 72). For those we utilized a Welch’s t-test. Asterisks denote statistical significances as listed above in legend.

To assess deficits in visual motor sensory processing we conducted the light dark locomotor activity (visuomotor response, VMR) assay at 6 dpf following heat shock induction of dnVDR at either 24, 48, or 72 hpf. Results of this analysis demonstrate that induction of the dominant negative vitamin D receptor within these time periods altered behavioral outcomes across four swimming parameters including distance moved, velocity, activity state, and distance to center point of the well (Fig 1A–L and Table 1). Induction of the dnVDRa at 24 hpf results in significant reductions in the dark phase total distance moved (p < 0.0001), velocity (p < 0.001) and activity state (p < 0.01). Induction of the dnVDRa at 24 hpf also significantly reduced the light phase total distance moved (p < 0.0001) and velocity (p < 0.001), while increasing distance to point (p < 0.01). The second timepoint of dnVDRa induction, 48 hpf, caused similar changes in the dark phase for total distance moved (p < 0.01), velocity (p < 0.05) and activity state (p < 0.05). Induction of the dnVDRa at 48 hpf also resulted in significant alterations in the light phase in total distance moved (p < 0.05), velocity (p < 0.01) and distance to point (p < 0.01). The final timepoint of dnVDRa induction, 72 hpf, resulted in the least severe effects with alterations only in the light phase in total distance moved (p < 0.05), activity state (p < 0.05) and velocity (p < 0.05). These results indicate that disruption of vitamin D receptor signaling via induction of the dnVDRa at early timepoints at 24 and 48 hpf has severe impacts on light dark locomotor activity.

**Table 1:** Visuo-motor response (VMR) assay at 6 dpf.

Parameter	Distance Moved (mm)				Velocity (mm/s)				Activity State (frequency)				Distance Moved (mm)			
Bin	Dark		Light		Dark		Light		Dark		Light		Dark		Light	
Data	Avg.	SD	Avg.	SD	Avg.	SD	Avg.	SD	Avg.	SD	Avg.	SD	Avg.	SD	Avg.	SD
**24-**	0.11	0.02	0.05	0.01	3.44	1.14	1.57	0.69	59.86	17.65	15.70	9.02	4.34	0.15	3.82	0.26
**24+**	**0.04**	**0.01**	**0.01**	**0.00**	**1.22**	**0.42**	**0.34**	**0.42**	**14.99**	**5.99**	**0.87**	**1.42**	4.26	0.08	**4.22**	**0.18**
**48-**	0.11	0.02	0.03	0.01	3.12	0.74	0.60	0.28	49.36	16.67	3.14	1.87	4.15	0.20	3.68	0.54
**48+**	**0.06**	**0.02**	**0.00**	**0.00**	**2.23**	**0.64**	**0.14**	**0.08**	**30.55**	**13.05**	**0.44**	**0.54**	4.10	0.15	**4.26**	**0.10**
**72-**	0.09	0.03	0.04	0.03	2.59	0.83	1.14	0.78	28.45	11.21	6.19	3.62	4.04	0.30	3.90	0.27
**72+**	0.08	0.02	**0.01**	**0.00**	2.27	0.44	**0.20**	**0.11**	39.08	12.84	**0.72**	**0.67**	3.85	0.40	4.01	0.31

All behavioral data values at 6 dpf. Data values for all dnVDRa induced timepoints (24 + , 48+ and 72+) and their controls (24-, 48-, 72-) for all VMR parameters. Bolded values represent statistical difference between the dnVDRa induced fish versus their controls. N = 8 for all groups in VMR Assay.

### Induction of dnVDR modulates early life stage acoustic startle response (ASR)

**Fig 2:**
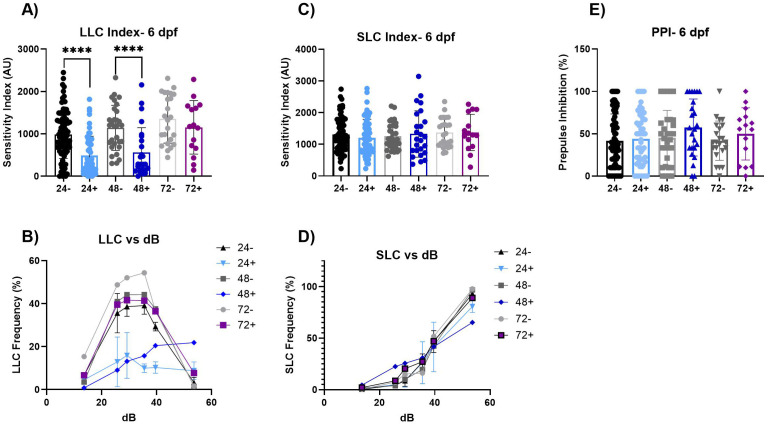
Acoustic startle at 6 dpf. (A-B) These graphs represent the area under the curve of the LLC response frequency. There are significant decreases in LLC responses in the 24+ (p < 0.0001) and 48+ (p ≤ 0.0001) fish. (C-D) These graphs represent the area under the curve of the SLC response frequency. There are no significant changes between groups for SLC response. (E) The percentage of PPI. We see no significant changes across the groups. Values represent the mean by separate measurements from individual fish: 24-: n = 70, 24 + : n = 54, 48-:31, 48 + : n = 25, 72-: n = 24 and 72 + : n = 15. Error bars represent the standard deviation of each group. All groups passed Normality tests except for the following: 24+ (LLC, PPI Inhibition), 24- (PPI Inhibition) and 48+ (LLC). An unpaired t-test was used for all groups exhibiting normality except for when variances were significantly different (SLC-48). For those we utilized a Welch’s t-test. For any group that failed Normality tests, a Mann-Whitney t-test was utilized. Asterisks denote statistical significances as listed above in legend.

**Fig 3:**
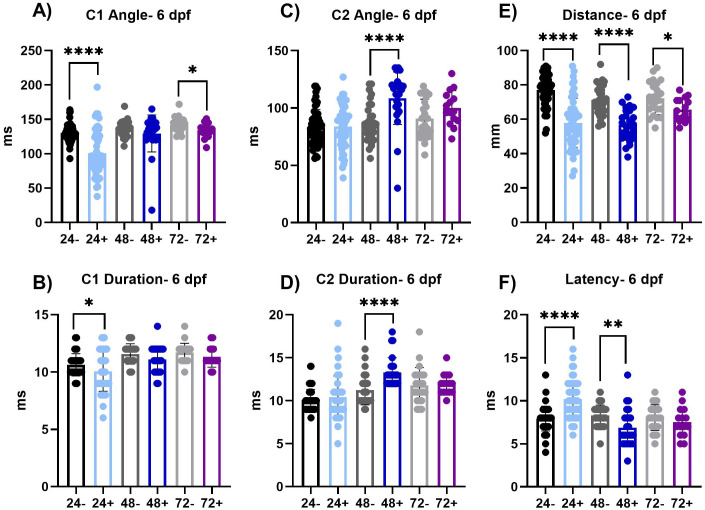
SLC response kinematics at 6 dpf. (A-B) C1 is the first C-bend performed. The angle and duration of the C1 bend is significantly decreased in 24 + fish (p < 0.0001 and p < 0.05) compared to the 24- fish and the C1 angle is decreased in 72 + fish (p < 0.05). (C-D) C2 is the second C-bend performed. The angle and duration of C2 bend is significantly increased in 48 + fish (p < 0.0001) compared to the 48- fish. (E-F) The total distance moved and latency, the total time of delay from the auditory stimulus, of the SLC response. The total distance of the SLC response is significantly decreased in 24 + fish, 48 + fish (p < 0.0001) and 72+ (p < 0.05) compared to their respective controls (24-, 48-, 72-). The latency is significantly increased in the 24 + fish (p < 0.0001) compared to the 24- fish and significantly decreased in the 48 + fish (p < 0.01) compared to the 48- fish. Values represent the mean by separate measurements of individual fish: 24-: n = 57, 24 + : n = 51−52, 48-:31, 48 + : n = 26, 72-: n = 24 and 72 + : n = 15. Error bars represent the standard deviation of each group. All groups passed Normality tests except for the following: 24+ (Distance, C1 angle), 24- (Distance, C1 angle), 48- (Distance), 48+ (Distance, C1 angle, C1 duration) and all groups failed for C1 and C2 duration. Any group that failed Normality tests, a Mann-Whitney t-test was utilized. An unpaired t-test was used for the rest of the groups except for when variances were significantly different (Distance-24). For those we utilized a Welch’s t-test. Asterisks denote statistical significances as listed above in legend.

To further identify alterations in larval zebrafish sensory processing, we next assessed the acoustic startle response with six-day old zebrafish larvae that have been subjected to dnVDR induction as described with the VMR assay. Results of this assay (**Fig 2** and **Table 2**) demonstrate a significant and specific impact on Long Latency C bends (LLC), quantified by calculating the area under the curve of stimulus intensity vs. LLC frequency (LLC index), post dnVDRa induction at both 24 and 48 hpf (p < 0.0001 and p < 0.0001, respectively). Conversely, we observed that Pre-pulse Inhibition (PPI) and the total Short Latency C bends (SLC) responses, measured by the SLC index, were unaffected across all dnVDR induction time points. The SLC responses can be categorized into a series of C-bends where the angles and durations of those bends can be quantified (S2 Fig). Kinematic assessment of SLC behaviors did reveal some significant alterations in the performance of these high-velocity escape responses at 24 and 48 hpf dnVDRa induction time points (**Fig 3**). For instance, the 24 hpf dnVDRa induced larvae exhibit a decrease in the initial C1 bend angle (p < 0.0001), reduced C1 bend duration (p < 0.05), increased overall latency (p < 0.0001) and a decrease in distance moved (p < 0.0001) for the total SLC response. The 48 hpf dnVDRa induced larvae exhibit an increased counterbend or C2 bend angle (p < 0.0001) and increased C2 bend duration (p < 0.0001), decreased overall latency (p < 0.001) and a decrease in distance moved (p < 0.0001) for the total SLC response as well. The 72 hpf dnVDRa induced larvae display a decrease in C1 bend angle (p < 0.05). These results indicate that while dnVDR induced larvae fish do perform SLC responses at normal frequencies, their performance of that response is significantly impaired which may indicate that other deficits are present. Representative images of 6 dfp and 28dpf C-bends are provided in S2 Fig.

**Table 2:** Acoustic startle responses (ASR) at 6 dpf.

Parameter	ASR						SLC Kinematics											
	LLC Index		PPI		SLC Index		C1 Angle (degrees)		C1 Duration (s)		C2 Angle (degrees)		C2 Duration (s)		Distance (mm)		Latency (s)	
Data	Avg.	SD	Avg.	SD	Avg.	SD	Avg.	SD	Avg.	SD	Avg.	SD	Avg.	SD	Avg.	SD	Avg.	SD
**24-**	982.84	558.38	41.44	31.76	1311.26	540.62	130.12	12.01	10.63	0.97	83.12	15.47	10.05	1.25	77.00	8.92	7.93	1.42
**24+**	**486.45**	**453.06**	43.89	30.66	1203.89	580.21	**100.78**	**32.51**	**10.06**	**1.72**	83.83	19.03	10.43	2.43	**57.96**	**14.11**	**10.21**	**2.13**
**48-**	1141.15	521.58	45.18	31.79	1255.77	422.76	136.77	10.67	11.58	0.87	87.48	16.33	11.23	1.68	71.32	8.49	8.29	1.20
**48+**	**560.50**	**573.05**	57.41	32.95	1325.04	717.36	129.50	26.40	11.12	1.12	**108.58**	**22.43**	**13.31**	**1.66**	**56.73**	**8.79**	**7.19**	**2.60**
**72-**	1354.74	561.43	41.75	22.65	1371.00	447.38	142.17	10.32	11.71	0.79	90.75	16.60	11.79	2.02	72.21	9.15	8.04	1.46
**72+**	1152.67	609.31	49.76	29.73	1372.97	553.88	134.07	10.34	11.33	0.87	100.00	14.57	11.93	1.18	**65.67**	**6.48**	7.53	1.67

All behavioral data values at 6 dpf. Data values for all dnVDRa induced timepoints (24 + , 48+ and 72+) and their controls (24-, 48-, 72-) for all ASR parameters. Bolded values represent statistical difference between the dnVDRa induced fish versus their controls. For ASR at 6 dpf: 24-: n = 70, 24 + : n = 54, 48-: n = 31, 48 + : n = 25, 72-: n = 24 and 72 + : n = 15.

### Induction of dnVDR results in persistent behavioral deficits in VMR and ASR assays

**Fig 4:**
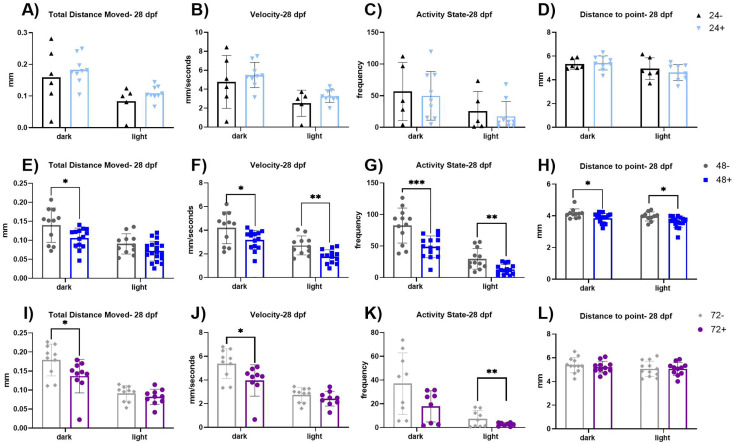
Visual motor response assay at 28 dpf. (A-D) VMR parameters in dnVDRa induced zebrafish at 24 hpf, there is no significant difference at 28 dpf. (E-H) VMR parameters in dnVDRa induced zebrafish at 48 hpf. There is a significant decrease in distance moved in the dark period (p < 0.05), a significant decrease in velocity in the dark (p < 0.05) and light (p < 0.01) periods, a significant decrease in activity state in the dark period (p < 0.001) and light period (p < 0.01) and a significant decrease in distance to point in the dark period (p < 0.05) and light period (p < 0.05) in the 48 + fish. (I-K) VMR parameters in dnVDRa induced zebrafish at 72 hpf. There is a significant decrease in distance moved in the dark period (p < 0.05), a significant decrease in velocity in the dark period (p < 0.05) and a significant decrease in activity state the light period (p < 0.01) in the 72 + fish. Values represent the mean by separate measurements in individual fish: 24-: n = 5-12, 24 + : n = 9, 48-: n = 10-12, 48 + : n = 15-19, 72-: n = 9-10 and 72 + : n = 8-10. Error bars represent the standard deviation of each group. All groups passed Normality tests except for the following: 72- (total distance moved-dark, velocity-dark), 48- (distance to point- dark & light) and 24+ (activity state-light). Any group that failed Normality tests, a Mann-Whitney t-test was utilized. An unpaired t-test was used for the rest of the groups except for when assessing activity state in the light period for the 24 and 72 heat shock groups as their variances were significantly different. For those we utilized a Welch’s t-test. Asterisks denote statistical significances as listed above in legend.

**Fig 5:**
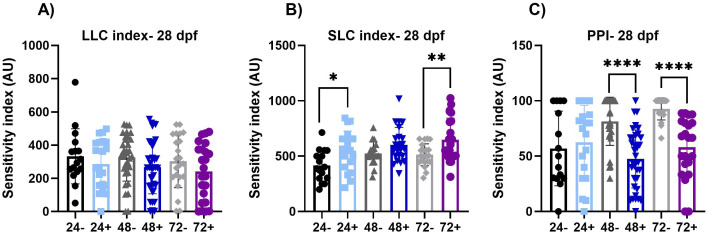
Acoustic Startle at 28 dpf. (A-B) These graphs represent the area under the curve of the SLC response frequency and LLC response frequency, respectively. There is a significant increase in SLC responses in the 24+ (p ≤ 0.05) and 72+ (p ≤ 0.01) fish. (C) There is a significant decrease of PPI in the 48+ and 72 + fish (p < 0.0001). Values represent the mean by separate measurements in individual fish: 24-: n = 16, 24 + :n = 21, 48-: n = 18, 48 + : n = 23, 72-: n = 22 and 72 + : n = 23. Error bars represent the standard deviation of each group. All groups passed Normality tests except for the following: 48- (PPI), 72- (PPI) and 72+ (LLC). Any group that failed Normality tests a Mann-Whitney t-test was utilized. An unpaired t-test was used for the rest of the groups except for when variances were significantly different (SLC-72). For those we utilized a Welch’s t-test. Asterisks denote statistical significances as listed above in legend.

**Fig 6:**
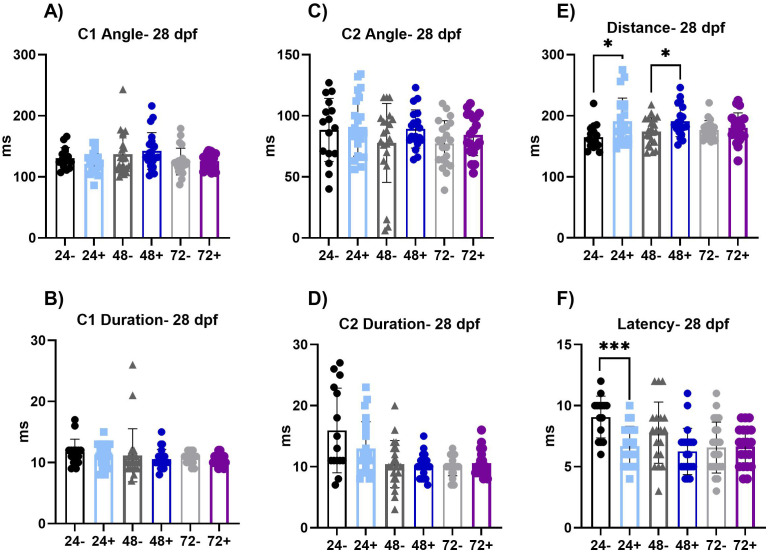
SLC response kinematics at 28 dpf. (A-B) C1 is the first C-bend performed. The angle and duration of the C1 bend is not significantly altered at this time point. (C-D) C2 is the second C-bend performed. The angle and duration of C2 bend is not significantly altered at this time point. (E-F) The total distance moved and latency, the total time of delay from the auditory stimulus, of the SLC response. The total distance of the SLC response is significantly increased in 24 + fish (p < 0.05) compared to 24- fish and in 72 + fish (p < 0.05) compared to 72- fish. The latency is significantly decreased in the 24 + fish (p < 0.001) and the 48 + fish (p < 0.05) compared to their respective controls (24- and 48-). Values represent the mean by separate measurements in individual fish: 24-: n = 15-16, 24 + : n = 23, 48-: n = 18-19, 48 + : n = 20-23, 72-: n = 21-23 and 72 + : n = 19-20. Error bars represent the standard deviation of each group. All groups passed Normality tests except for the following: 24-(C1 and C2 duration), 24+ (Distance), 48- (C1 angle and duration, C2 angle), 48+ (Latency, C1 duration), 72- (C1 angle and duration, C2 duration) and 72+ (C1 and C2 duration). Any group that failed Normality tests a Mann-Whitney t-test was utilized. An unpaired t-test was used for the rest of the groups except for when variances were significantly different (Distance-72, C2 duration-48). For those we utilized a Welch’s t-test. Asterisks denote statistical significances as listed above in legend.

To assess if induction of dnVDRa expression during early development can result in long term deficits we assessed both behavior assays at 28 dpf (Figs 4–6 and Tables 3,4) following a single heat shock treatment between 24–72 hpf. With heat shock at 24 hpf, results indicate that deficits observed in the light dark locomotor activity assay at 6 dpf do not persist through 28 dpf. Conversely, when dnVDR is induced at 48 hpf, persistent effects on VMR outcomes are observed at the 28 dpf assessment. These include alterations in total distance moved (p < 0.05 dark phase only), velocity (p < 0.05 and p < 0.01, dark and light phases respectively), activity state (p < 0.001 and p < 0.01, dark and light phases respectively), and distance to center point (p < 0.05 and p < 0.05, dark and light phases respectively). With induction of dnVDR occurring at 72 hpf and behavioral assessment conducted at 28dpf, we additionally observed persistent alterations in total distance moved (p < 0.05), velocity (p < 0.05) in the dark phases, and activity state (p < 0.01), in the light phase only.

**Table 3:** Visuo-motor response (VMR) assay at 28 dpf.

Parameter	Distance Moved (mm)				Velocity (mm/s)				Activity State (frequency)				Distance Moved (mm)			
Bin	Dark		Light		Dark		Light		Dark		Light		Dark		Light	
Data	Avg.	SD	Avg.	SD	Avg.	SD	Avg.	SD	Avg.	SD	Avg.	SD	Avg.	SD	Avg.	SD
**24-**	0.16	0.08	0.11	0.07	4.77	2.54	3.32	2.12	56.59	41.20	25.59	27.97	5.32	0.39	4.95	0.84
**24+**	0.18	0.04	0.11	0.02	5.50	1.25	3.27	0.63	49.87	36.29	17.45	22.03	5.43	0.56	4.62	0.63
**48-**	0.14	0.04	0.09	0.03	4.21	1.29	2.72	0.76	82.51	26.68	29.64	15.62	4.15	0.28	3.96	0.27
**48+**	**0.11**	**0.03**	0.07	0.03	**3.17**	**0.77**	**1.78**	**0.55**	**48.83**	**16.63**	**12.74**	**7.51**	**3.85**	**0.29**	**3.62**	**0.34**
**72-**	0.18	0.04	0.09	0.02	5.37	1.19	2.74	0.58	37.10	24.36	7.48	6.40	5.40	0.61	5.07	0.62
**72+**	**0.14**	**0.04**	0.08	0.02	**3.96**	**1.25**	2.43	0.59	17.88	12.29	**2.73**	**1.34**	5.21	0.48	5.06	0.52

All behavioral data values at 28 dpf. Data values for all dnVDRa induced timepoints (24 + , 48+ and 72+) and their controls (24-, 48-, 72-) for all VMR parameters. Bolded values represent statistical difference between the dnVDRa induced fish versus their controls. For VMR: 24-: n = 6, 24 + : n = 9, 48-: n = 12, 48 + : n = 15, 72-: n = 9 and 72 + : n = 8.

**Table 4:** Acoustic startle responses (ASR) at 28 dpf.

Parameter	ASR						SLC Kinematics											
	LLC Index		PPI		SLC Index		C1 Angle (degrees)		C1 Duration (s)		C2 Angle (degrees)		C2 Duration (s)		Distance (mm)		Latency (s)	
Data	Avg.	SD	Avg.	SD	Avg.	SD	Avg.	SD	Avg.	SD	Avg.	SD	Avg.	SD	Avg.	SD	Avg.	SD
**24-**	332.63	160.68	56.97	32.59	415.53	136.12	130.73	16.61	11.69	2.08	88.38	24.99	15.93	6.69	164.69	19.85	9.07	1.65
**24+**	286.54	144.08	62.47	32.54	**552.31**	**170.71**	121.05	17.13	11.30	1.76	90.82	23.35	12.95	4.31	**190.86**	**37.16**	**6.70**	**1.57**
**48-**	325.82	140.26	81.53	21.38	524.59	101.11	137.43	33.75	11.19	4.24	77.71	31.54	10.43	3.76	174.05	21.97	7.79	2.44
**48+**	266.35	157.49	**47.50**	**25.93**	602.80	153.94	142.33	29.32	10.59	1.53	89.20	15.24	10.19	1.84	**190.80**	**24.45**	6.25	1.84
**72-**	303.15	156.44	92.74	9.68	532.96	133.34	125.00	21.31	10.82	0.89	77.24	18.23	10.10	1.54	176.67	14.94	6.57	2.04
**72+**	241.31	165.14	**58.01**	**26.45**	**649.49**	**189.61**	125.75	12.36	10.50	0.81	84.47	16.68	10.58	2.09	180.00	23.91	6.65	1.59

All behavioral data values at 28 dpf. Data values for all dnVDRa induced timepoints (24 + , 48+ and 72+) and their controls (24-, 48-, 72-) for all ASR parameters. Bolded values represent statistical difference between the dnVDRa induced fish versus their controls. For ASR: 24-: n = 16, 24 + : n = 21, 48-: n = 18, 48 + : n = 23, 72-: n = 22 and 72 + : n = 23.

We did not observe significant alterations in LLC index at 28 dpf (**Fig 5A**) with dnVDRa induced in zebrafish embryos at any early developmental stage (24, 48 or 72 hpf). We did however observe an increase in SLC index in the 24 hpf dnVDRa induced fish (p < 0.05) and the 72 hpf dnVDRa induced fish (p < 0.01). We did not observe persistent alterations in the C-bends when we analyzed SLC kinematics at 28 dpf (**Fig 6**). We did however observe an increase in distance moved in the 24 hpf dnVDRa induced fish (p < 0.05) and 72 hpf dnVDR (p < 0.05). There also was a decrease in latency in both 24 and 72 hpf dnVDRa induced fish (p < 0.001 and p < 0.05, 24+ and 48 + respectively) in the SLC response. We also observed a significant decrease in pre-pulse inhibition (PPI) in our 48 hpf dnVDRa induced fish (p < 0.0001) and 72 hpf dnVDRa induced fish (p < 0.001) when assayed at 28 dpf (Fig 5C). Pre-pulse inhibition (PPI) is a key form of sensorimotor plasticity in which a weak pre-pulse reduces responses to subsequent strong stimuli. Interestingly PPI was unaffected when early-stage embryos were induced and assayed at 6dpf (**Fig 2E**). However, as with the VMR assay, 28-day-old young adult fish that received a single heat shock at 48 hpf displayed significantly reduced PPI, indicating dysfunction in sensory processing later in development.

### Neurotransmitter gene expression at 6 dpf

**Fig 7:**
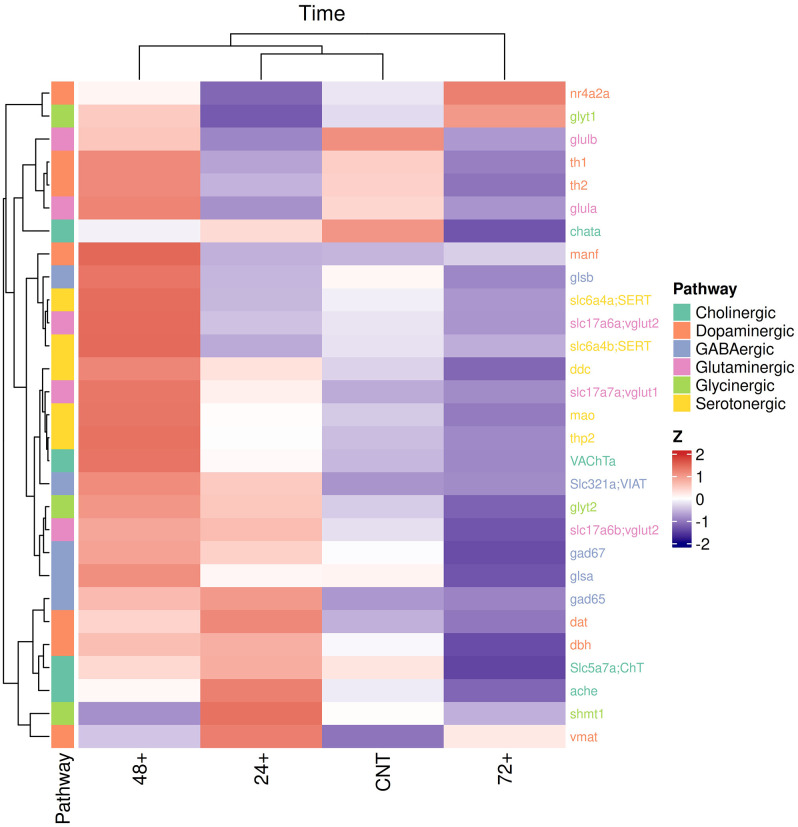
Consolidated gene expression of neurotransmitter pathways. Heat map showing the row Z-score, a scaling method to compare all values in the row, for gene expression in neurotransmitter genes at 6 dpf in dnVDRa induced fish at either 24, 48 or 72 hpf and the control group. Higher gene expression values are indicated in red while lower values are in blue. The key depicts genes belong to each pathway. Values represent the mean from individual fish with 3-4 fish/group.

Next, we examined expression of genes associated with six select neurotransmitter pathways with known roles in sensory function; cholinergic, glycinergic, serotonergic, GABAergic, glutaminergic and dopaminergic. Results from this assessment indicate significant alterations in expression of multiple genes across each dnVDRa induction period and within all pathways examined (S3 Fig). To visualize global gene expression events within these pathways, gene expression was formatted into a heat map using hierarchical clustering (**Fig 7**). Gene expression data resulted in three empirical clusters, with C-I comprised of the 48hpf dnVDRa induction period, the C-II comprised of two sub clusters including the 24hpf induction period and the control group and C-III comprised of the 72 hpf induction period. Results indicate that gene expression with the 48 hpf dnVDRa induced fish was significantly different from the 24 dnVDRa induced fish, which are more similar to the control group.

## Discussion

The nervous system can distinguish between different sensory stimuli, integrate multiple simultaneous stimuli, and initiate the appropriate behavioral response in specific contexts. Specifically, in vertebrates visual, tactile, and auditory stimuli all elicit stereotyped behaviors [40–43]. These behavioral responses are modulated by specific parameters associated with each stimuli including luminance, size, and speed (visual) [44–46] or intensity (tactile, auditory) [43,47]. The importance of proper sensory processing and proper behavioral responses can be highlighted by the findings that disruptions in sensory responsiveness are observed in many neuropsychiatric disorders including anxiety [48–51], PTSD [52], ADHD [53], migraine [54], schizophrenia [55,56], and autism [57–59]. The molecular and cellular mechanisms that drive the development of the neural circuits regulating sensorimotor responsiveness are complex and incompletely understood.

In this study we observe that modulation of VDR signaling during early zebrafish neurodevelopment results in attenuation of sensory processing activities. Specifically, we observe alterations in defined locomotion activities within our light/dark (VMR) assay and the ability to perform and execute Long Latency C-bend (LLC) response and Short Latency C-bend (SLC) responses in our acoustic startle response assay. These studies demonstrate that early embryonic disruptions in vitamin D signaling modulate sensory processing activities with lasting effects in juvenal forms consistent with a Developmental Origins of Health and Disease (DOHaD) [26,60] model of neurodevelopment.

The VMR assay is a well-established test that measures swimming behaviors in response to whole-field light/dark stimuli [61,62]. For zebrafish larvae, transition from light to dark results in increased locomotion (velocity, distance moved, mobility, activity state, and angular velocity), typically attributed to a stressed- or anxiety-like state, while transition from dark to light results in decreased locomotion. This behavioral plasticity requires proper functioning of visual and motor systems, as well as integration centers in the brain that connect visual perception, internal state, and locomotor function [63]. Overall, our assessments suggest that modulation of VDR signaling through induction of a dominant form of VDR (dnVDR) at 24 or 48 hpf resulted in marked decrease in both activity state and hypolocomotion in both light and dark phases of our locomotor activity assay at 6 dpf. When we assess this behavior at 28 dpf, we demonstrate that dnVDRa induction at 48 or 72 hpf results in a significant decrease in all the tested locomotor parameters. Induction of the dnVDRa at 48 hpf caused the most severe and persistent behavioral phenotypes, eliciting sustained hypolocomotion in both light and dark phases at 28 dpf. This reduced locomotion in the light-dark transitions suggest that early developmental modulation of VDR signaling leads to impaired sensorimotor integration. Interestingly, contrary to our findings, studies in rodent models have demonstrated hyperlocomotion with developmental vitamin D deficiency [64]. Our model, however, utilizes a conditional dominant negative VDR knock down system that enables temporal regulation of VDR and behavior assessment at very early stages of organismal development compared to rodent models of VDD. Overall, our findings confirm previous observations from our group that dietary modulations or environmental exposures to xenobiotics that modulate developmental VDR signaling results in altered behavioral function [65,66].

We next tested zebrafish startle responses using an acoustic startle response assay. In this assay zebrafish are exposed to an abrupt acoustic stimulus and can respond with one of two stereotyped behaviors either Short-Latency C-bends (SLCs) or Long-Latency C-bends (LLCs). These responses have been identified by multiple kinematic parameters such as latency, turn angle, and pectoral fin involvement. SLCs are typically performed when zebrafish are exposed to intense acoustic stimuli and are initiated within 4–15 ms. Weaker acoustic stimuli typically trigger LLCs and are initiated 15–100 ms after the stimulus, are more varied in terms of kinematics, and can be influenced by visual input [67,68]. Additionally, these behaviors have been demonstrated to have differing neural circuitry. The SLC response is triggered by Mauthner neurons (M-cells), which receive direct input from auditory afferents and contact spinal motor neurons [38,68,69]. LLC responses occur independent of M-cell function and instead depend on a bilateral population of pre-pontine neurons located between the locus coeruleus and cerebellum [68]. It has been hypothesized that due to the delayed response, LLC behaviors may allow for integration of multimodal stimuli and context-appropriate decision-making [68].

In this study we find that early developmental induction of dnVDR between 24–48 hpf in zebrafish embryos causes a severe reduction in LLC responses but relatively little effect on SLC responses. These data suggest that VDR signaling selectively impacts LLC and not SLC circuit function, as all groups are able to perform SLC reactions at normal rates. However, our data also indicate that dnVDRa induction at 24 or 48 hpf alters SLC kinematics at 6 dpf. Specifically, dnVDRa induction at 24 hpf reduces C1 bend angle and duration while dnVDRa induction at 48 hpf increases C2 bend angle and duration. Induction at both 24 and 48 hpf reduced the distance traveled during SLC responses at 6 dpf, and so together these findings indicate that while VDR signaling has minimal impacts on SLC initiation, it is important for the fine motor control of these responses, likely affecting spinal motor circuits. Conversely, the long-term effects of developmental dnVDR induction on the acoustic startle response in 28 dpf fish were mostly reversed. LLCs were unaffected while SLCs were increased in the 24 hpf and 72 hpf groups. We also observed pronounced deficits in pre-pulse inhibition when dnVDRa is induced at 48 or 72 hpf and fish assayed at 28dpf. Pre-pulse inhibition (PPI) is a neurological phenomenon in which a weak pre-pulse reduces responses to subsequent strong stimuli and can be related to sensorimotor plasticity. PPI can also be a measure for sensorimotor gating, which can assess the brain’s ability to filter out irrelevant sensory information and prevent an excessive response to stimuli. PPI has become important in the context of neuropsychiatric disorders and could be a potential biomarker of brain function [69,70]. Deficits in PPI are a common endophenotype of schizophrenia [55,56], so this finding is consistent with the association of VDD with neuropsychiatric condition [71,72] and further supports the importance of investigating the underlying mechanisms. The deficits in PPI at 28 dpf may indicate an inability to properly modulate startle responses later in development. With these behavioral assays we demonstrate that dnVDRa induction at 48 hpf results in the most severe behavior phenotypes that are sustained to 28 dpf, highlighting the importance of VDR signaling during development for both visually driven locomotor activity and acoustically-evoked behaviors and behavioral modulation. Together our behavior data reveal that developmental disruption of VDR signaling causes multiple yet specific defects in neural function (e.g., LLCs but not SLCs affected). This suggests that the defects are unlikely to be secondary to systemic effects of VDR dysfunction but are rather the result of the loss of VDR function in the underlying neural circuits. This is further reinforced by the fact that the dnVDR is largely expressed in the brain and spinal cord, with comparatively little expression in non-neural tissues (S1 Fig).

To coordinate behavior phenotypes with putative molecular changes caused by dnVDR induction, we examined gene expression in multiple neurotransmitter pathways in the context of development. In addition to driving all sensory and motor function, neurotransmitter signaling impacts cell proliferation, migration, and differentiation during brain development [73–79]. Vitamin D is also well-known to modulate synaptic function by altering the expression of receptors, transporters and the enzymes involved in metabolism and synthesis of neurotransmitters [13,64,27,80,81]. Previous developmental VDD rat models have demonstrated disruptions to dopaminergic, glutamatergic and serotonergic pathways [21,64,27,80,82–85]. Specifically, it has been demonstrated that VDR activation can result in induction in expression of tryptophan hydroxylase 2 (THP2) which is the rate limiting enzyme in the synthesis of serotonin [27] and has been found to repress expression of monoamine oxidase-A (MAO) and serotonin reuptake transporter (SERT) at certain concentrations *in vitro* [83]. These trends indicate vitamin D modulates the serotonergic system to result in increased serotonin levels. In the dopaminergic system, VDR overexpression results in elevated catechol-o-methyl transferase (COMT), the crucial enzyme in dopamine synthesis and *in vivo* vitamin D-deficient rats show reductions in COMT [21] indicating VDR signaling is important for dopamine synthesis. Within the inhibitory systems (GABA, glutaminergic), dietary vitamin D deficient rodent models identified that low vitamin D levels results in decreased expression of glutamate and GABA transporters (GAT) [84]. Additionally, in an adult vitamin D deficient mouse model, expression of glutamic acid decarboxylase (GAD65/67) an important enzyme in GABA synthesis was found to be reduced [80]. These studies illustrate that VDR signaling is involved in both excitatory and inhibitory pathways in the brain. In this study we focused on key components of cholinergic, GABAergic, glycinergic, glutamatergic, serotonergic, and dopaminergic neurotransmitter pathways at 6 dpf in all dnVDR induced groups compared to our non-induced dnVDR controls. Our data demonstrate that induction of dnVDR modulates expression of essential genes within these pathways. Expression differed dependent upon the timing of dnVDRa induction. Consistent with our behavior data, dnVDRa induction at 48 hpf resulted in gene expression changes that were most different from control groups and the other induction time points when analyzed through hierarchical clustering. These data are consistent with the current findings in mammalian models of VDD and illustrate the importance of temporal disruptions in vitamin D signaling and modulation of essential neurotransmitter pathways. These broad impacts likely contribute to the persistent behavior phenotypes observed in this study.

These studies provide insight on how developmental VDR signaling modulates neural gene expression and behavior. In conjunction, mammalian model systems of developmental VDD illustrate distinct behavioral phenotypes such as significant alterations in social behavior, learning and memory. As such, current research supports the hypothesis that developmental vitamin D deficiency adversely impacts brain development resulting in modulation of adult behaviors consistent with a DOHaD model of VDD [26,60]. Yet a significant gap remains in our understanding of both the mechanisms of VDD on DOHaD and windows of susceptibility to VDD.

## Supporting information

S1 Table Primer sequences for neurotransmitter related qPCR. Primer sequences for neurotransmitter related qPCR. Gene expression was normalized to *efla*, the reference gene.
(DOCX)

S1 Fig BFP expression in 48 hpf heat shocked transgenic zebrafish. Epifluorescent images of TG(hsp:dnVDRa:BFP) heat shocked at 48 hpf and imaged 5 hours post heat shock. We can see fish heat shocked but very minimal BFP expression which are deemed 48-. The dnVDRa positive fish, 48 + , have distinct BFP expression in the cranial region.
(JPG)

S2 Fig Representative images of ASR assay. Representative example of Short-Latency C-Bend (SLC) in 6 dpf and 28 dpf zebrafish. Composite images show the initial C-bend (C1) (Red arrow) and counterbend (C2) (Blue arrow) during the first 40 ms of the response. 6 dpf: scale bar 2 mm; 28 dpf: scale bar 5 mm.
(JPG)

S3 Fig Gene expression of neurotransmitter pathways at 6 dp. A. Gene expression of neurotransmitter pathways at 6 dpf, Glycinergic pathway gene expression. In the 24 hpf dnVDRa induced group we see a significant decrease in glyt1 expression (p < 0.05) and an increase in glyt2 expression (p < 0.05) and in slc32a1 expression (p < 0.01). In the 48 hpf dnVDRa induced fish there is a significant increase in glyt2 and slc32a1 expression (p < 0.05). In the 72 hpf dnVDRa induced fish there is a significant decrease in glyt2 expression (p < 0.01). Values represent the mean by separate measurements in individual fish with 3–4 fish per gene. Error bars represent the standard deviation of each group. An unpaired t-test with a Welch’s t-test was used for groups when variances were significantly different. Asterisks denote statistical significances as listed above in legend. B. Gene expression of neurotransmitter pathways at 6 dpf, Cholinergic pathway gene expression. There are no altered genes in this panel for the 24 hpf dnVDRa induced fish. In the 48 hpf dnVDRa induced fish there is a significant increase in VAChTa expression (p < 0.001). In the 72 hpf dnVDRa induced fish there is a significant decrease in glyt2 expression (p < 0.05). Values represent the mean by separate measurements in individual fish with 3–4 fish per gene. Error bars represent the standard deviation of each group. An unpaired t-test with a Welch’s t-test was used for groups when variances were significantly different. Asterisks denote statistical significances as listed above in legend. C. Gene expression of neurotransmitter pathways at 6 dpf, Serotonergic pathway gene expression. In the 24 hpf dnVDRa induced group we see a significant decrease in slc6a4b expression (p < 0.05). In the 48 hpf dnVDRa induced fish there is a significant increase in ddc (p < 0.05), thp2 (p < 0.01), mao (p < 0.05) and slc6a4b (p < 0.01) expression. In the 72 hpf dnVDRa induced fish there is a significant decrease in mao (p < 0.01) and slc6a4b (p < 0.05) expression). Values represent the mean by separate measurements in individual fish with 3–4 fish per gene. Error bars represent the standard deviation of each group. An unpaired t-test with a Welch’s t-test was used for groups when variances were significantly different. Asterisks denote statistical significances as listed above in legend. D. Gene expression of neurotransmitter pathways at 6 dpf, GABAergic pathway gene expression. In the 24 hpf dnVDRa induced group we see a significant increase in gad67 expression (p < 0.05). In the 48 hpf dnVDRa induced fish there is a significant increase in gad65 (p < 0.01) and gad67 (p < 0.05) expression. In the 72 hpf dnVDRa induced fish there is a significant decrease in gad67 (p < 0.05) and glsa (p < 0.05) expression. Values represent the mean by separate measurements in individual fish with 3–4 fish per gene. Error bars represent the standard deviation of each group. An unpaired t-test with a Welch’s t-test was used for groups when variances were significantly different. Asterisks denote statistical significances as listed above in legend. E. Gene expression of neurotransmitter pathways at 6 dpf, Glutaminergic pathway gene expression. In the 24 hpf dnVDRa induced group we see a significant increase in slc17a6b (p < 0.05) expression). In the 48 hpf dnVDRa induced fish there is a significant increase in slc17a7a (p < 0.01), slc17a6a (p < 0.01) and slc17a6b (p < 0.05) expression. In the 72 hpf dnVDRa induced fish there is a significant decrease in glsa (p < 0.05), glula (p < 0.01), glulb (p < 0.05) and slc17a6a (p < 0.05) expression. Values represent the mean by separate measurements in individual fish with 3–4 fish per gene. Error bars represent the standard deviation of each group. An unpaired t-test with a Welch’s t-test was used for groups when variances were significantly different. Asterisks denote statistical significances as listed above in legend. F. Gene expression of neurotransmitter pathways at 6 dpf, Dopaminergic pathway gene expression. In the 24 hpf dnVDRa induced group we see a significant increase in dat and vmat expression (p < 0.05) and a decrease in th1 (p < 0.05), th2 (p < 0.01) and nr4a2a (p < 0.05) expression. In the 48 hpf dnVDRa induced fish there is a significant increase in dbh (p < 0.01), dat (p < 0.05) and manf (p < 0.001) expression. In the 72 hpf dnVDRa induced fish there is a significant decrease in dbh (p < 0.01) and th2 (p < 0.05) expression. Values represent the mean by separate measurements in individual fish with 3–4 fish per gene. Error bars represent the standard deviation of each group. An unpaired t-test with a Welch’s t-test was used for groups when variances were significantly different. Asterisks denote statistical significances as listed above in legend.
(ZIP)
