# Supplementary material for: Early disruptions in vitamin D receptor signaling induces persistent developmental behavior deficits in zebrafish larvae
Source: PLoS One. 2025 Nov 14;20(11):e0335156. doi: 10.1371/journal.pone.0335156 (PMC12617856; doi:10.1371/journal.pone.0335156)
Supplement: S1 Table — Primer sequences for neurotransmitter related qPCR. Gene expression was normalized to efla, the reference gene. (DOCX) [file pone.0335156.s001.docx]

**S1 Table. Primer sequences for neurotransmitter related qPCR**. Primer sequences for neurotransmitter related qPCR. Gene expression was normalized to *efla*, the reference gene

| Gene | **Primer Sequence (5’- 3’)** | **Product Size (bp**) |
| --- | --- | --- |
| ache | TCCTGCTTTTGCTTTTCGCC (forward) | 245 |
|  | AACGTGAGTAGCACAGTGGG (reverse) |  |
| chata | ACGAGTCGACAACATTCGCT (forward) | 114 |
|  | ATGGCAGTCCAGAGCAACTC (reverse) |  |
| dat | CTAAAAAGCTCCGCATCCAG (forward) | 231 |
|  | TGTCCAAGAGCAAAGCAATG (reverse) |  |
| dbh | TGGCCTATCATATCCCGCTG (forward) | 209 |
|  | CCACGCATCCCCAAAATAGG (reverse) |  |
| ddc | ACTGGCACAGCCCATACTTC (forward) | 169 |
|  | AGCATCTTTCCCAGCCAGTC (reverse) |  |
| ef1a | TACAAATGCGGTGGAATCGAC (forward) | 246 |
|  | GTCAGCCTGAGAAGTACCAGT (reverse) |  |
| gad65 | ATGGTGCCTTTGATCCTTTG (forward) | 228 |
|  | TCTGCATCAGTCCCTCCTCT (reverse) |  |
| gad67 | GACGACAAGGGTCGAATTGT (forward) | 211 |
|  | TGCGCACGTAGTTAGTGAGG (reverse) |  |
| glsa | TTTAGGCTTTCGAGGGCGTT (forward) | 165 |
|  | GCCTGCCTTCCTTCTCTTGT (reverse) |  |
| glsb | TGTGGACTAAAGTGTCGCCC (forward) | 175 |
|  | ACGTGACCCGAATGAGAACC (reverse) |  |
| glula | TGCCTCAGGGAGACCAAGTA (forward) | 158 |
|  | GCCTTCAGCTTGATACGTGC (reverse) |  |
| glulb | ATATCAGGCTGAGGGGTCCA (forward) | 272 |
|  | GAGGTCCAGGGAAGCCATTG (reverse) |  |
| glyt1 | TGAGCACTCATCGTGCCAAT (forward) | 242 |
|  | GCCTGATGACTTGACTCCCC (reverse) |  |
| glyt2 | TTCCAGGACGATGATGATGA (forward) | 237 |
|  | GAACTGACCCAACGACACCT (reverse) |  |
| manf | AGCGGCTTTACCAGACGATA (forward) | 149 |
|  | CTTTGTTGCTGCATCACTCG (reverse) |  |
| mao | TCTGGCACGAAAATCACGGA (forward) | 103 |
|  | TAACGCCTCCTCTGATCCCA (reverse) |  |
| nr4a2a | AGTATGGCTCATCTCCGCAA (forward) | 209 |
|  | CGGCTTCACGTCATAACTGG (reverse) |  |
| shmt1 | CTTCTGGCAGACATGGCTCA (forward) | 280 |
|  | GCTTCAAAGCAACAGCGACA (reverse) |  |
| slc17a6a | GCCACTCTGCTGTTAGTGGT (forward) | 184 |
|  | TACCAGAAAGTGTGCCGACC (reverse) |  |
| slc17a6b | TCCATGCCCGTCTATGCAATC (forward) | 205 |
|  | TTTTGCTGCGAAGGTGATCG (reverse) |  |
| slc17a7a | CGGCTCATTCTTCTGGGGTT (forward) | 166 |
|  | GACCATGATCACACACCCGT (reverse) |  |
| slc32a1 | CGGACAAGCCCAGAATCACT (forward) | 228 |
|  | CGTACGAGTCTCTCACTCGC (reverse) |  |
| slc5a7a | GAAGCTGGTGGACTCCTGAT (forward) | 262 |
|  | TGTCCCCTGTGAGATAAAAATTCCC (reverse) |  |
| slc6a4a | CTCCAGGCTCTCTATCCCCT (forward) | 188 |
|  | CGGAACGCTATCCACAACCA (reverse) |  |
| slc6a4b | AGCACTTTTGGTGGGTTGGA (forward) | 229 |
|  | AGGAGACTGCTGTTGCTTCC (reverse) |  |
| th1 | AGTGTGAAGTGCACCTGTCG (forward) | 233 |
|  | GGCAATGTCTCCGATCATCT (reverse) |  |
| th2 | CCCAGTACATTCGTCACCCT (forward) | 238 |
|  | GCCCCATAAGCCTTTACTGC (reverse) |  |
| thp2 | AGCCCCTAAATCACCTGTTGG (forward) | 180 |
|  | TTAAAGGATGTCCTGCGGAGC (reverse) |  |
| VAChT | GCCGAGGCTTCACTATTCAG (forward) | 223 |
|  | GTCCTGGATCGCATTTCCTA (reverse) |  |
| vmat | TCTTTCTCCATCGAGCACCT (forward) | 202 |
|  | GGCAAACAACAGACCCACTT (reverse) |  |
