# Supplementary figures and images for: Early disruptions in vitamin D receptor signaling induces persistent developmental behavior deficits in zebrafish larvae
Source: PLoS One. 2025 Nov 14;20(11):e0335156. doi: 10.1371/journal.pone.0335156 (PMC12617856; doi:10.1371/journal.pone.0335156)

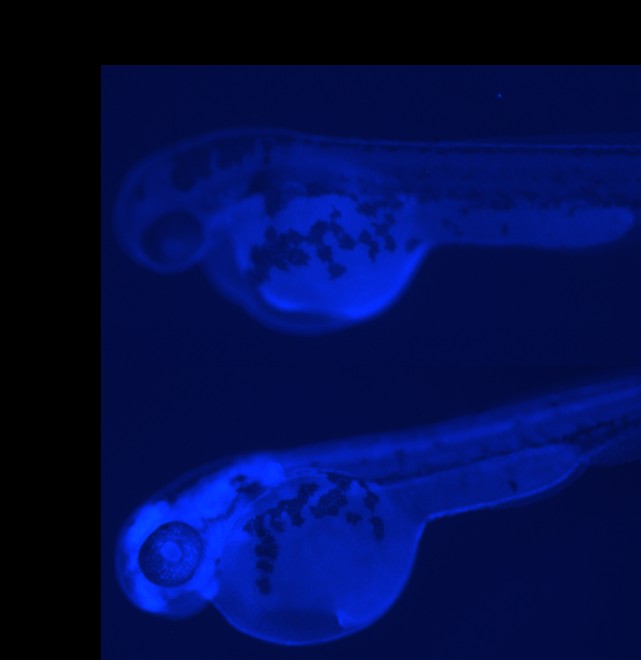

Supplement: S1 Fig — Epifluorescent images of TG(hsp:dnVDRa:BFP) heat shocked at 48 hpf and imaged 5 hours post heat shock. We can see fish heat shocked but very minimal BFP expression which are deemed 48-. The dnVDRa positive fish, 48 + , have distinct BFP expression in the cranial region. (JPG) [file pone.0335156.s002.jpg]

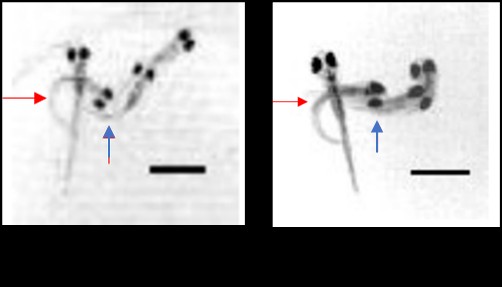

Supplement: S2 Fig — Representative example of Short-Latency C-Bend (SLC) in 6 dpf and 28 dpf zebrafish. Composite images show the initial C-bend (C1) (Red arrow) and counterbend (C2) (Blue arrow) during the first 40 ms of the response. 6 dpf: scale bar 2 mm; 28 dpf: scale bar 5 mm. (JPG) [file pone.0335156.s003.jpg]

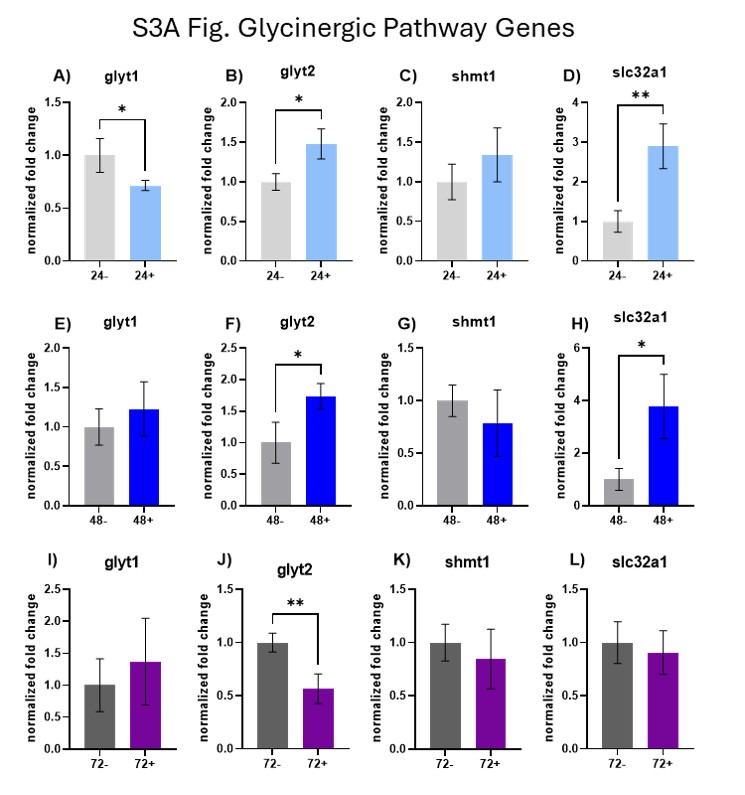

Supplement: S3 Fig — A. Gene expression of neurotransmitter pathways at 6 dpf, Glycinergic pathway gene expression. In the 24 hpf dnVDRa induced group we see a significant decrease in glyt1 expression (p < 0.05) and an increase in glyt2 expression (p < 0.05) and in slc32a1 expression (p < 0.01). In the 48 hpf dnVDRa induced fish there is a significant increase in glyt2 and slc32a1 expression (p < 0.05). In the 72 hpf dnVDRa induced fish there is a significant decrease in glyt2 expression (p < 0.01). Values represent the mean by separate measurements in individual fish with 3–4 fish per gene. Error bars represent the standard deviation of each group. An unpaired t-test with a Welch’s t-test was used for groups when variances were significantly different. Asterisks denote statistical significances as listed above in legend. B. Gene expression of neurotransmitter pathways at 6 dpf, Cholinergic pathway gene expression. There are no altered genes in this panel for the 24 hpf dnVDRa induced fish. In the 48 hpf dnVDRa induced fish there is a significant increase in VAChTa expression (p < 0.001). In the 72 hpf dnVDRa induced fish there is a significant decrease in glyt2 expression (p < 0.05). Values represent the mean by separate measurements in individual fish with 3–4 fish per gene. Error bars represent the standard deviation of each group. An unpaired t-test with a Welch’s t-test was used for groups when variances were significantly different. Asterisks denote statistical significances as listed above in legend. C. Gene expression of neurotransmitter pathways at 6 dpf, Serotonergic pathway gene expression. In the 24 hpf dnVDRa induced group we see a significant decrease in slc6a4b expression (p < 0.05). In the 48 hpf dnVDRa induced fish there is a significant increase in ddc (p < 0.05), thp2 (p < 0.01), mao (p < 0.05) and slc6a4b (p < 0.01) expression. In the 72 hpf dnVDRa induced fish there is a significant decrease in mao (p < 0.01) and slc6a4b (p < 0.05) expression). Values represent the [file pone.0335156.s004.zip › S3A Fig.jpg]

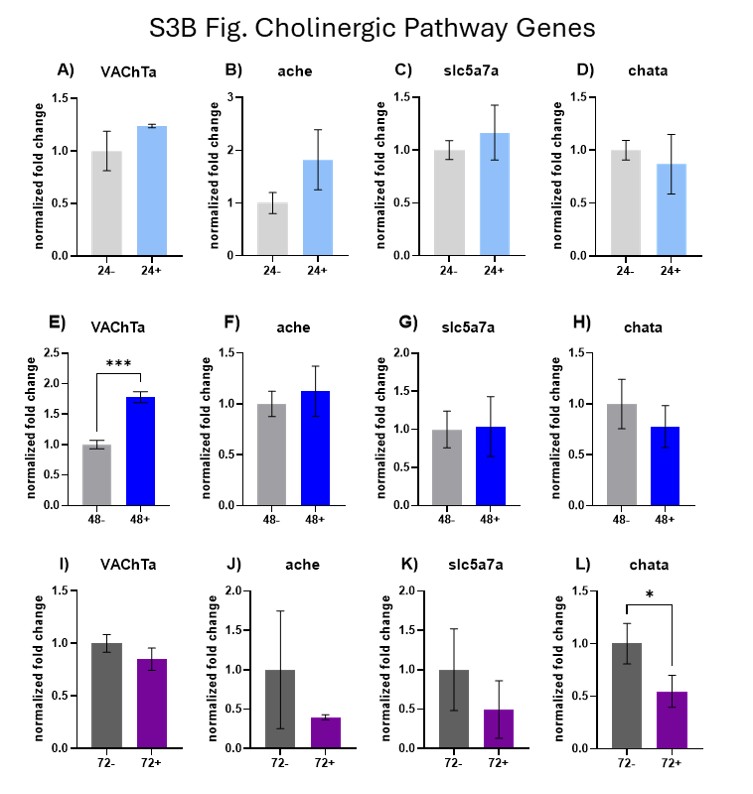

Supplement: S3 Fig — A. Gene expression of neurotransmitter pathways at 6 dpf, Glycinergic pathway gene expression. In the 24 hpf dnVDRa induced group we see a significant decrease in glyt1 expression (p < 0.05) and an increase in glyt2 expression (p < 0.05) and in slc32a1 expression (p < 0.01). In the 48 hpf dnVDRa induced fish there is a significant increase in glyt2 and slc32a1 expression (p < 0.05). In the 72 hpf dnVDRa induced fish there is a significant decrease in glyt2 expression (p < 0.01). Values represent the mean by separate measurements in individual fish with 3–4 fish per gene. Error bars represent the standard deviation of each group. An unpaired t-test with a Welch’s t-test was used for groups when variances were significantly different. Asterisks denote statistical significances as listed above in legend. B. Gene expression of neurotransmitter pathways at 6 dpf, Cholinergic pathway gene expression. There are no altered genes in this panel for the 24 hpf dnVDRa induced fish. In the 48 hpf dnVDRa induced fish there is a significant increase in VAChTa expression (p < 0.001). In the 72 hpf dnVDRa induced fish there is a significant decrease in glyt2 expression (p < 0.05). Values represent the mean by separate measurements in individual fish with 3–4 fish per gene. Error bars represent the standard deviation of each group. An unpaired t-test with a Welch’s t-test was used for groups when variances were significantly different. Asterisks denote statistical significances as listed above in legend. C. Gene expression of neurotransmitter pathways at 6 dpf, Serotonergic pathway gene expression. In the 24 hpf dnVDRa induced group we see a significant decrease in slc6a4b expression (p < 0.05). In the 48 hpf dnVDRa induced fish there is a significant increase in ddc (p < 0.05), thp2 (p < 0.01), mao (p < 0.05) and slc6a4b (p < 0.01) expression. In the 72 hpf dnVDRa induced fish there is a significant decrease in mao (p < 0.01) and slc6a4b (p < 0.05) expression). Values represent the [file pone.0335156.s004.zip › S3B Fig.jpg]

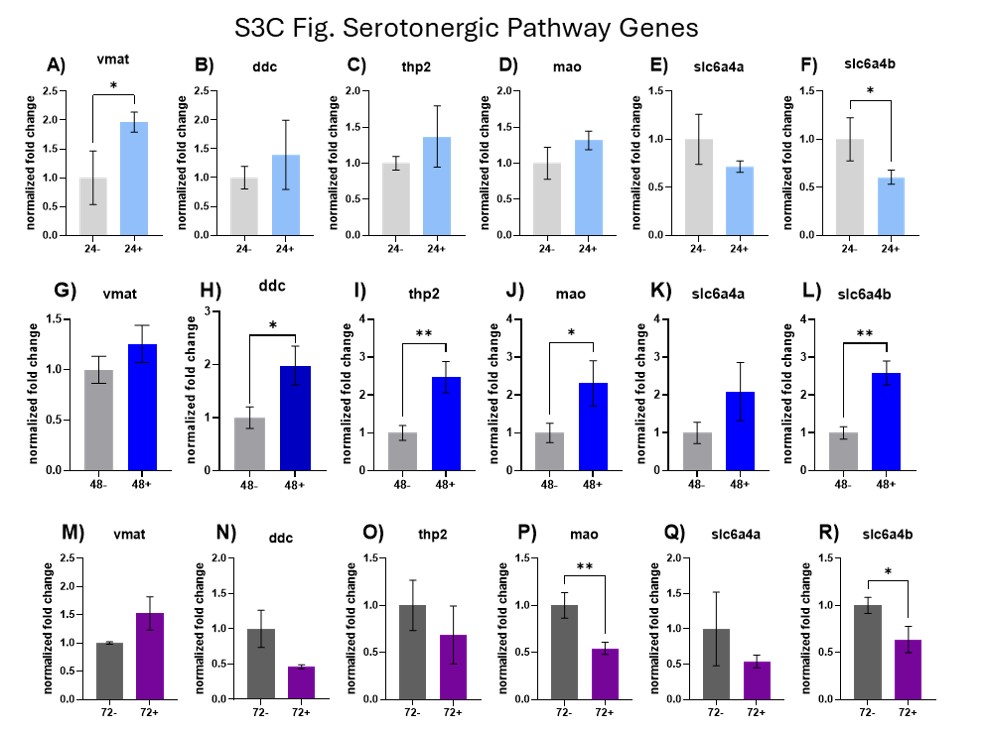

Supplement: S3 Fig — A. Gene expression of neurotransmitter pathways at 6 dpf, Glycinergic pathway gene expression. In the 24 hpf dnVDRa induced group we see a significant decrease in glyt1 expression (p < 0.05) and an increase in glyt2 expression (p < 0.05) and in slc32a1 expression (p < 0.01). In the 48 hpf dnVDRa induced fish there is a significant increase in glyt2 and slc32a1 expression (p < 0.05). In the 72 hpf dnVDRa induced fish there is a significant decrease in glyt2 expression (p < 0.01). Values represent the mean by separate measurements in individual fish with 3–4 fish per gene. Error bars represent the standard deviation of each group. An unpaired t-test with a Welch’s t-test was used for groups when variances were significantly different. Asterisks denote statistical significances as listed above in legend. B. Gene expression of neurotransmitter pathways at 6 dpf, Cholinergic pathway gene expression. There are no altered genes in this panel for the 24 hpf dnVDRa induced fish. In the 48 hpf dnVDRa induced fish there is a significant increase in VAChTa expression (p < 0.001). In the 72 hpf dnVDRa induced fish there is a significant decrease in glyt2 expression (p < 0.05). Values represent the mean by separate measurements in individual fish with 3–4 fish per gene. Error bars represent the standard deviation of each group. An unpaired t-test with a Welch’s t-test was used for groups when variances were significantly different. Asterisks denote statistical significances as listed above in legend. C. Gene expression of neurotransmitter pathways at 6 dpf, Serotonergic pathway gene expression. In the 24 hpf dnVDRa induced group we see a significant decrease in slc6a4b expression (p < 0.05). In the 48 hpf dnVDRa induced fish there is a significant increase in ddc (p < 0.05), thp2 (p < 0.01), mao (p < 0.05) and slc6a4b (p < 0.01) expression. In the 72 hpf dnVDRa induced fish there is a significant decrease in mao (p < 0.01) and slc6a4b (p < 0.05) expression). Values represent the [file pone.0335156.s004.zip › S3C Fig.jpg]

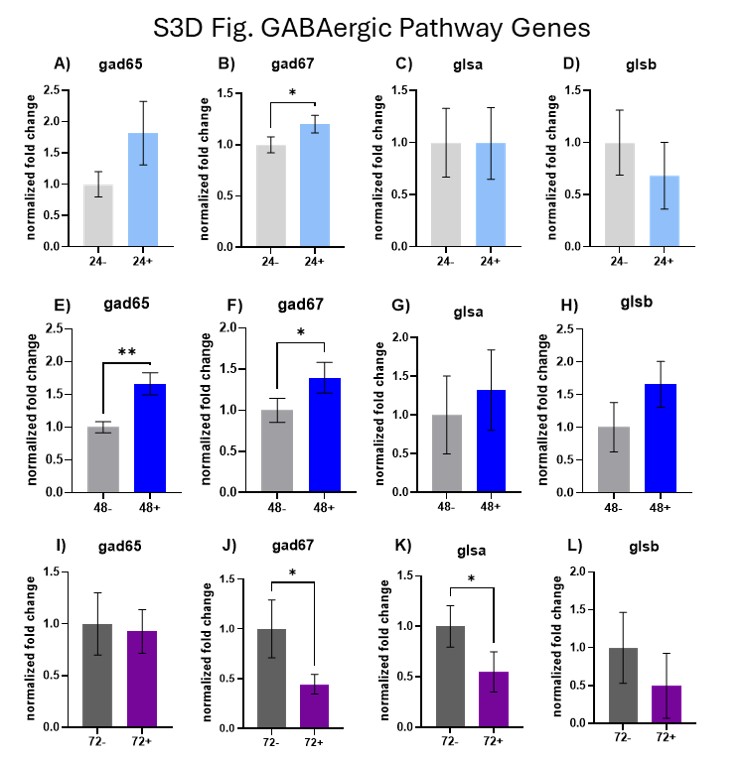

Supplement: S3 Fig — A. Gene expression of neurotransmitter pathways at 6 dpf, Glycinergic pathway gene expression. In the 24 hpf dnVDRa induced group we see a significant decrease in glyt1 expression (p < 0.05) and an increase in glyt2 expression (p < 0.05) and in slc32a1 expression (p < 0.01). In the 48 hpf dnVDRa induced fish there is a significant increase in glyt2 and slc32a1 expression (p < 0.05). In the 72 hpf dnVDRa induced fish there is a significant decrease in glyt2 expression (p < 0.01). Values represent the mean by separate measurements in individual fish with 3–4 fish per gene. Error bars represent the standard deviation of each group. An unpaired t-test with a Welch’s t-test was used for groups when variances were significantly different. Asterisks denote statistical significances as listed above in legend. B. Gene expression of neurotransmitter pathways at 6 dpf, Cholinergic pathway gene expression. There are no altered genes in this panel for the 24 hpf dnVDRa induced fish. In the 48 hpf dnVDRa induced fish there is a significant increase in VAChTa expression (p < 0.001). In the 72 hpf dnVDRa induced fish there is a significant decrease in glyt2 expression (p < 0.05). Values represent the mean by separate measurements in individual fish with 3–4 fish per gene. Error bars represent the standard deviation of each group. An unpaired t-test with a Welch’s t-test was used for groups when variances were significantly different. Asterisks denote statistical significances as listed above in legend. C. Gene expression of neurotransmitter pathways at 6 dpf, Serotonergic pathway gene expression. In the 24 hpf dnVDRa induced group we see a significant decrease in slc6a4b expression (p < 0.05). In the 48 hpf dnVDRa induced fish there is a significant increase in ddc (p < 0.05), thp2 (p < 0.01), mao (p < 0.05) and slc6a4b (p < 0.01) expression. In the 72 hpf dnVDRa induced fish there is a significant decrease in mao (p < 0.01) and slc6a4b (p < 0.05) expression). Values represent the [file pone.0335156.s004.zip › S3D Fig.jpg]

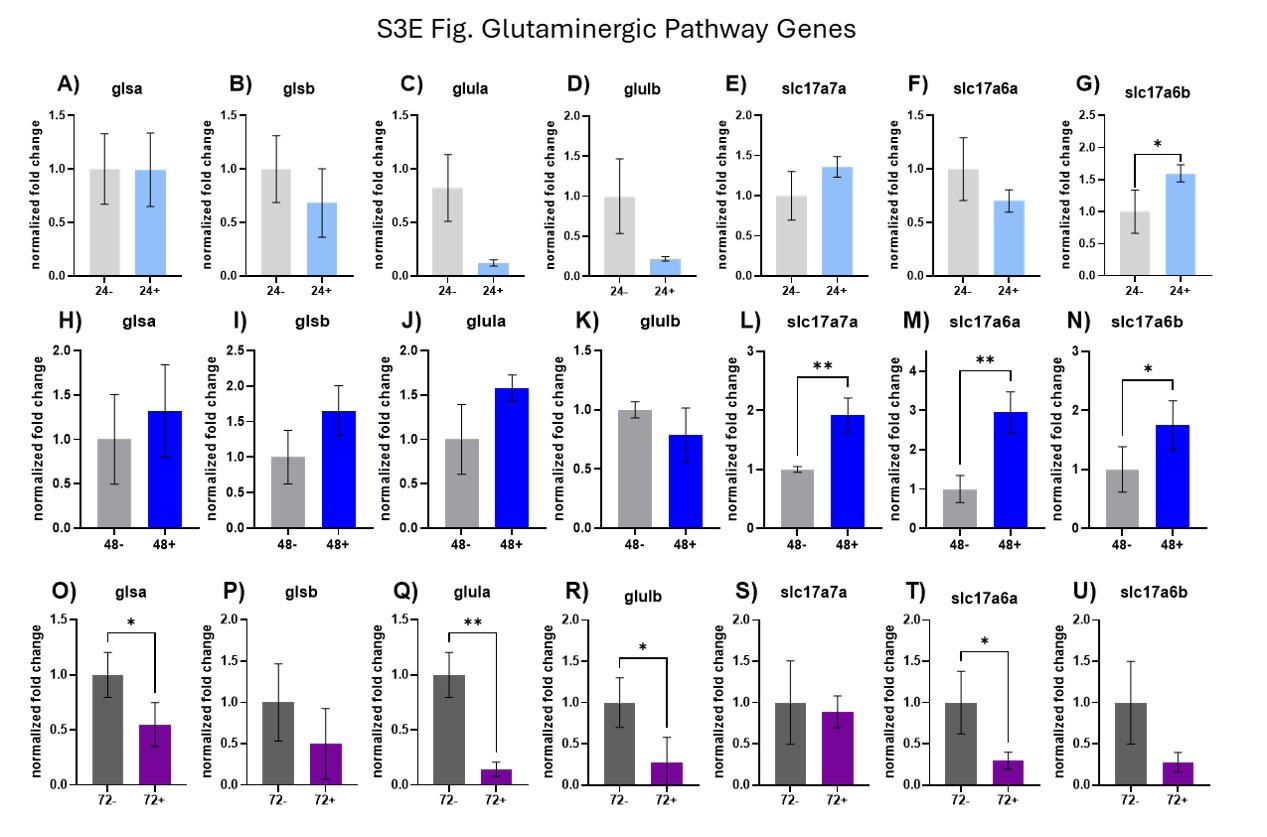

Supplement: S3 Fig — A. Gene expression of neurotransmitter pathways at 6 dpf, Glycinergic pathway gene expression. In the 24 hpf dnVDRa induced group we see a significant decrease in glyt1 expression (p < 0.05) and an increase in glyt2 expression (p < 0.05) and in slc32a1 expression (p < 0.01). In the 48 hpf dnVDRa induced fish there is a significant increase in glyt2 and slc32a1 expression (p < 0.05). In the 72 hpf dnVDRa induced fish there is a significant decrease in glyt2 expression (p < 0.01). Values represent the mean by separate measurements in individual fish with 3–4 fish per gene. Error bars represent the standard deviation of each group. An unpaired t-test with a Welch’s t-test was used for groups when variances were significantly different. Asterisks denote statistical significances as listed above in legend. B. Gene expression of neurotransmitter pathways at 6 dpf, Cholinergic pathway gene expression. There are no altered genes in this panel for the 24 hpf dnVDRa induced fish. In the 48 hpf dnVDRa induced fish there is a significant increase in VAChTa expression (p < 0.001). In the 72 hpf dnVDRa induced fish there is a significant decrease in glyt2 expression (p < 0.05). Values represent the mean by separate measurements in individual fish with 3–4 fish per gene. Error bars represent the standard deviation of each group. An unpaired t-test with a Welch’s t-test was used for groups when variances were significantly different. Asterisks denote statistical significances as listed above in legend. C. Gene expression of neurotransmitter pathways at 6 dpf, Serotonergic pathway gene expression. In the 24 hpf dnVDRa induced group we see a significant decrease in slc6a4b expression (p < 0.05). In the 48 hpf dnVDRa induced fish there is a significant increase in ddc (p < 0.05), thp2 (p < 0.01), mao (p < 0.05) and slc6a4b (p < 0.01) expression. In the 72 hpf dnVDRa induced fish there is a significant decrease in mao (p < 0.01) and slc6a4b (p < 0.05) expression). Values represent the [file pone.0335156.s004.zip › S3E Fig.jpg]

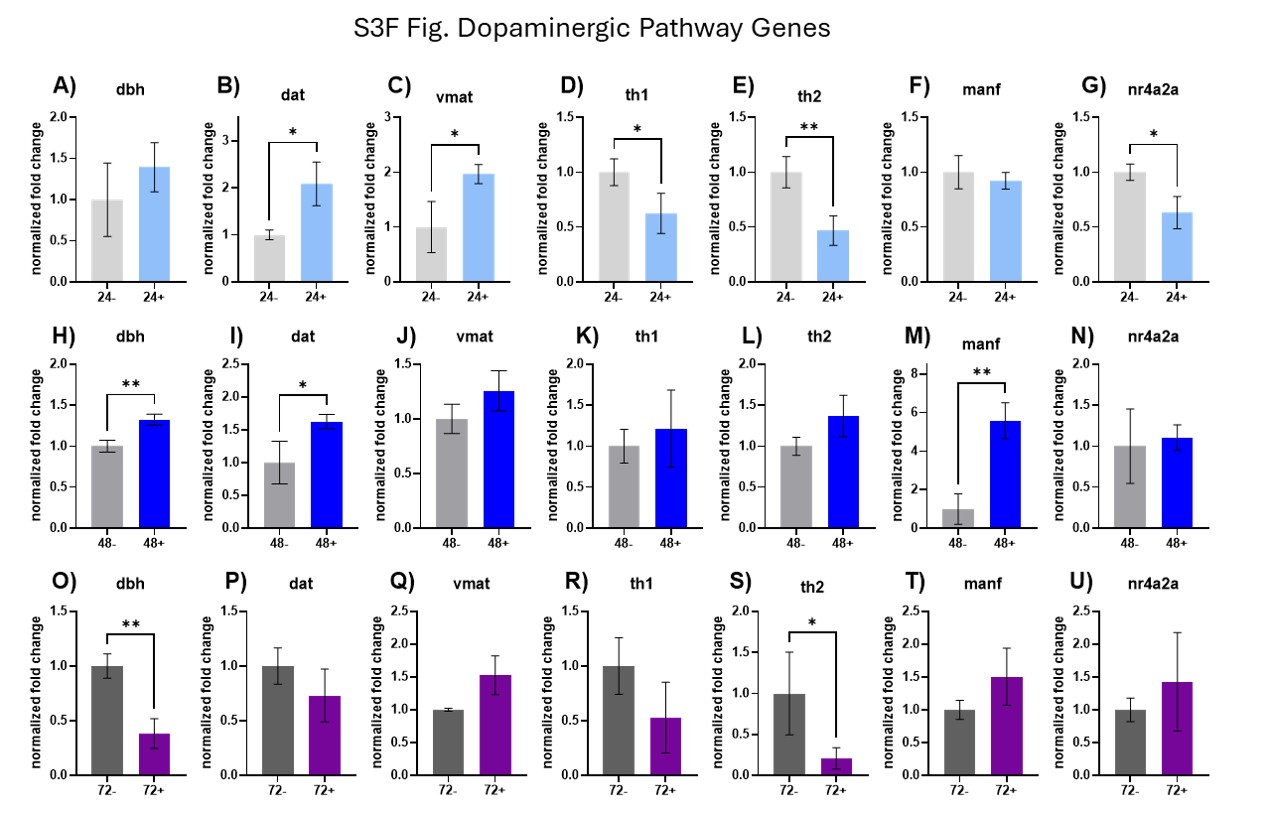

Supplement: S3 Fig — A. Gene expression of neurotransmitter pathways at 6 dpf, Glycinergic pathway gene expression. In the 24 hpf dnVDRa induced group we see a significant decrease in glyt1 expression (p < 0.05) and an increase in glyt2 expression (p < 0.05) and in slc32a1 expression (p < 0.01). In the 48 hpf dnVDRa induced fish there is a significant increase in glyt2 and slc32a1 expression (p < 0.05). In the 72 hpf dnVDRa induced fish there is a significant decrease in glyt2 expression (p < 0.01). Values represent the mean by separate measurements in individual fish with 3–4 fish per gene. Error bars represent the standard deviation of each group. An unpaired t-test with a Welch’s t-test was used for groups when variances were significantly different. Asterisks denote statistical significances as listed above in legend. B. Gene expression of neurotransmitter pathways at 6 dpf, Cholinergic pathway gene expression. There are no altered genes in this panel for the 24 hpf dnVDRa induced fish. In the 48 hpf dnVDRa induced fish there is a significant increase in VAChTa expression (p < 0.001). In the 72 hpf dnVDRa induced fish there is a significant decrease in glyt2 expression (p < 0.05). Values represent the mean by separate measurements in individual fish with 3–4 fish per gene. Error bars represent the standard deviation of each group. An unpaired t-test with a Welch’s t-test was used for groups when variances were significantly different. Asterisks denote statistical significances as listed above in legend. C. Gene expression of neurotransmitter pathways at 6 dpf, Serotonergic pathway gene expression. In the 24 hpf dnVDRa induced group we see a significant decrease in slc6a4b expression (p < 0.05). In the 48 hpf dnVDRa induced fish there is a significant increase in ddc (p < 0.05), thp2 (p < 0.01), mao (p < 0.05) and slc6a4b (p < 0.01) expression. In the 72 hpf dnVDRa induced fish there is a significant decrease in mao (p < 0.01) and slc6a4b (p < 0.05) expression). Values represent the [file pone.0335156.s004.zip › S3F Fig.jpg]
